# Hot Tub Lung: A Diagnostic Challenge

**DOI:** 10.7759/cureus.1617

**Published:** 2017-08-27

**Authors:** Hassaan Yasin, William E Mangano, Paras Malhotra, Ali Farooq, Hesham Mohamed

**Affiliations:** 1 Internal Medicine, West Virginia University - Charleston Division; 2 Department of Pathology, West Virginia University - Charleston Division

**Keywords:** hot tub lung, mycobacterium avium complex, cavitary lung nodules, hypersensitivity pneumonitis, nontuberculous mycobacterium

## Abstract

Hot tub lung (HTL) is a granulomatous lung disease thought to occur as a result of a hypersensitivity response to non-tuberculous mycobacteria (NTM). Typical radiographic findings are diffuse micronodular and/or ground glass opacities. We report an interesting case of HTL that presented with unique radiographic features, making its diagnosis a predicament. A 56-year-old immunocompetent female with chronic dyspnea and dry cough was found to have subcentimeter cavitary nodules, predominantly in the upper lung zones. Tissue culture obtained via bronchoscopy was positive for Mycobacterium avium complex (MAC). The patient’s clinical and radiographic status, however, deteriorated on antimycobacterial therapy. Complete clinical and radiographic resolution was achieved only after avoidance of hot tub use and treatment with steroids. We believe this is the first reported case of HTL manifesting as cavitary lung nodules with mediastinal lymphadenopathy, and we recommend physicians keep HTL in consideration when encountering patients with these radiographic findings.

## Introduction

Hot tub lung (HTL) is a diffuse granulomatous lung disease that occurs as a result of exposure to non-tuberculous mycobacteria (NTM) [1]. Typical radiographic features that have been reported so far in the literature are diffuse micronodular and/or ground-glass opacities [2]. Herein, we report a case of HTL with unique radiographic manifestations that made its diagnosis a predicament.

## Case presentation

A 56-year-old female was admitted to the hospital for a right hallux amputation for an infected diabetic foot ulcer. On review of systems, she reported progressive exertional shortness of breath for several months, accompanied by a productive cough with clear sputum. No fever, hemoptysis, or weight loss was reported. She was a former smoker with a past medical history of coronary artery disease, chronic obstructive pulmonary disease, and type 2 diabetes mellitus. Physical examination was remarkable for diffuse rhonchi with decreased breath sounds bilaterally on lung auscultation. 

**Figure 1:**
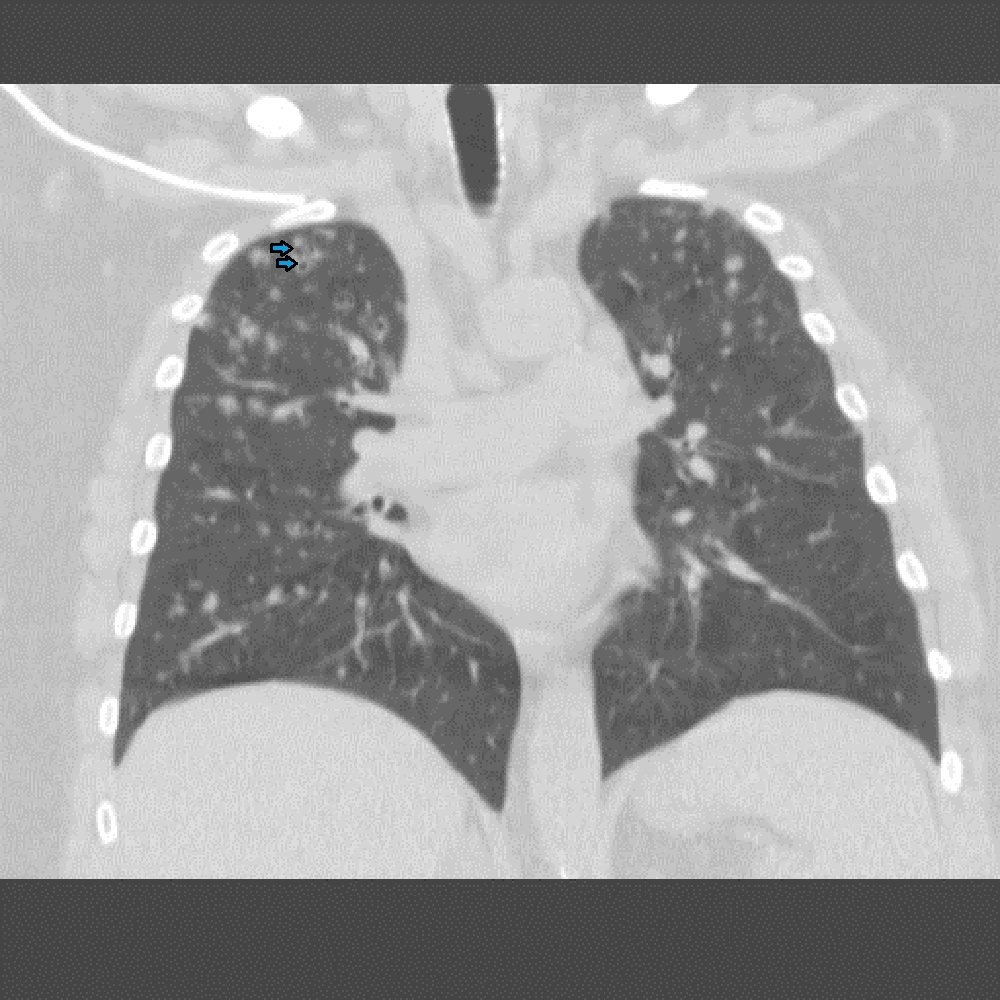
Computed tomography of the chest on initial evaluation demonstrating subcentimeter cavitary nodular lesions

**Figure 2:**
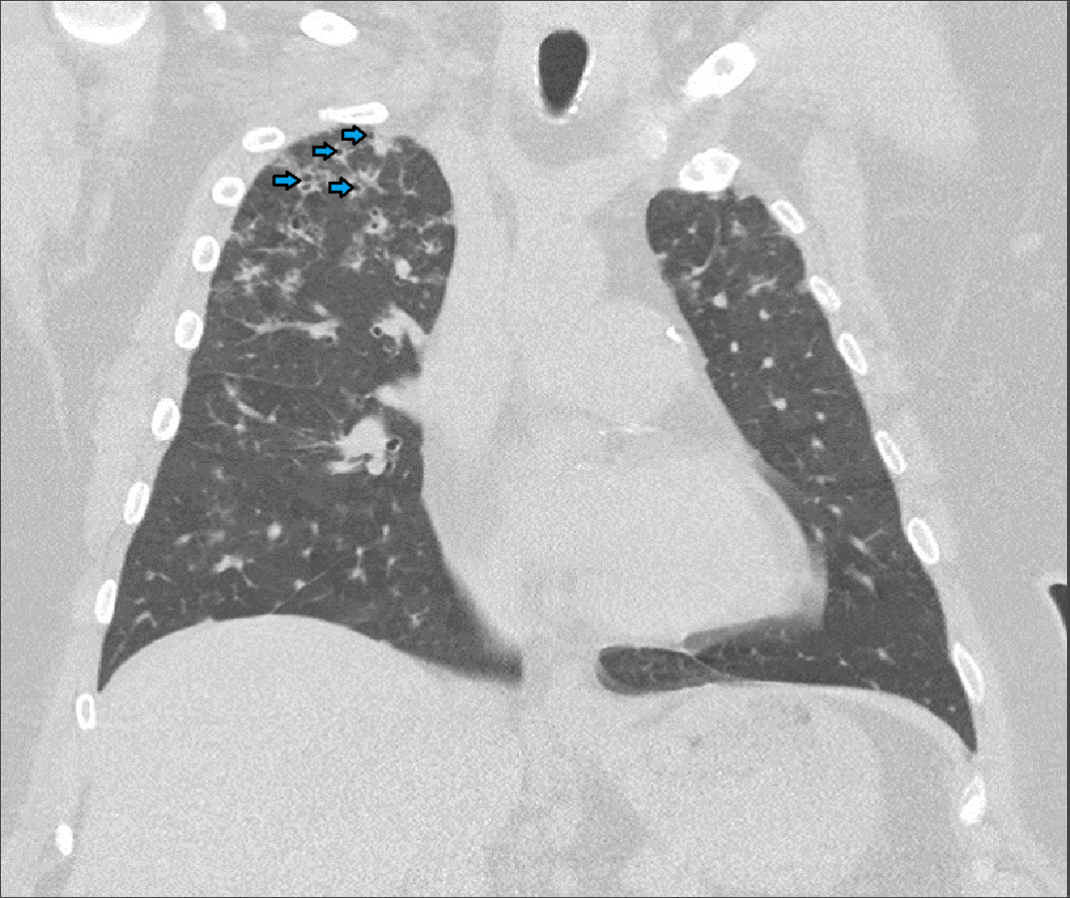
Computed tomography of the chest after three months of antimycobacterial therapy showing development of more cavitary lesions

**Figure 3:**
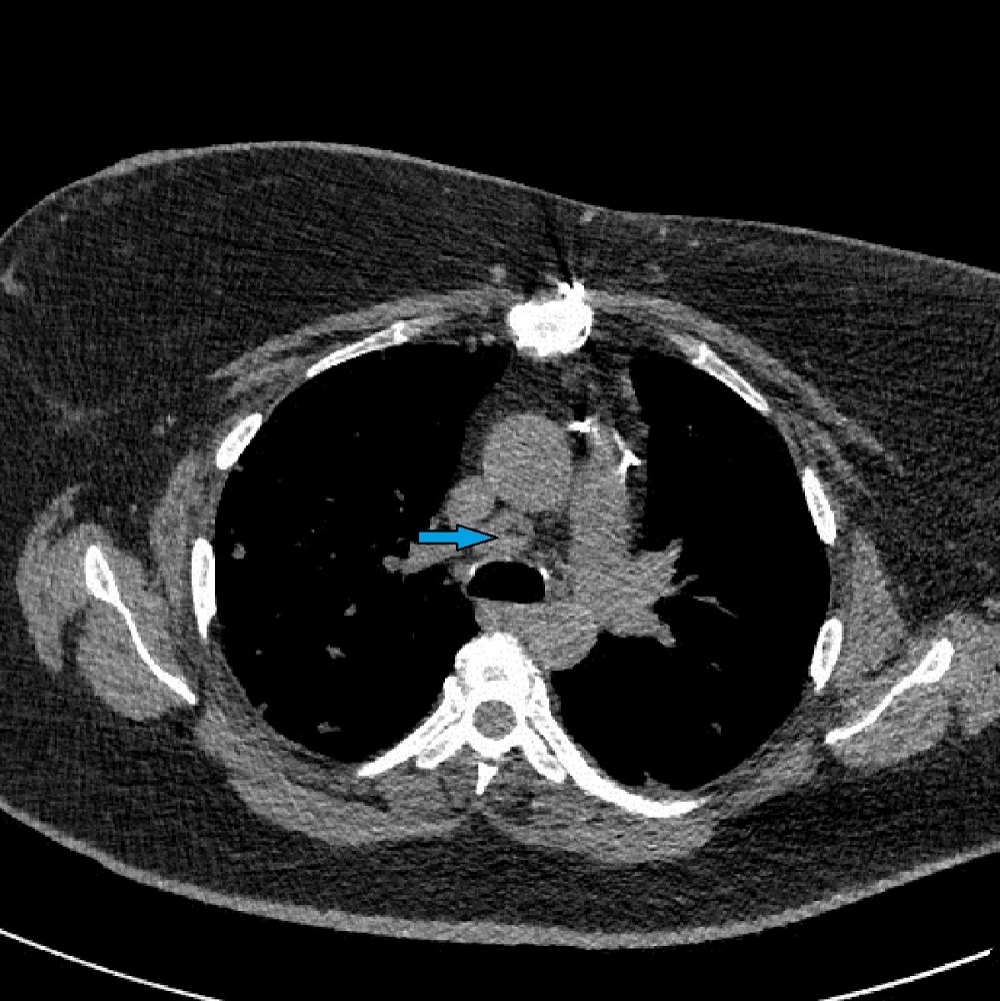
Computed tomography of the chest with mediastinal window showing an enlarged mediastinal lymph node

**Figure 4:**
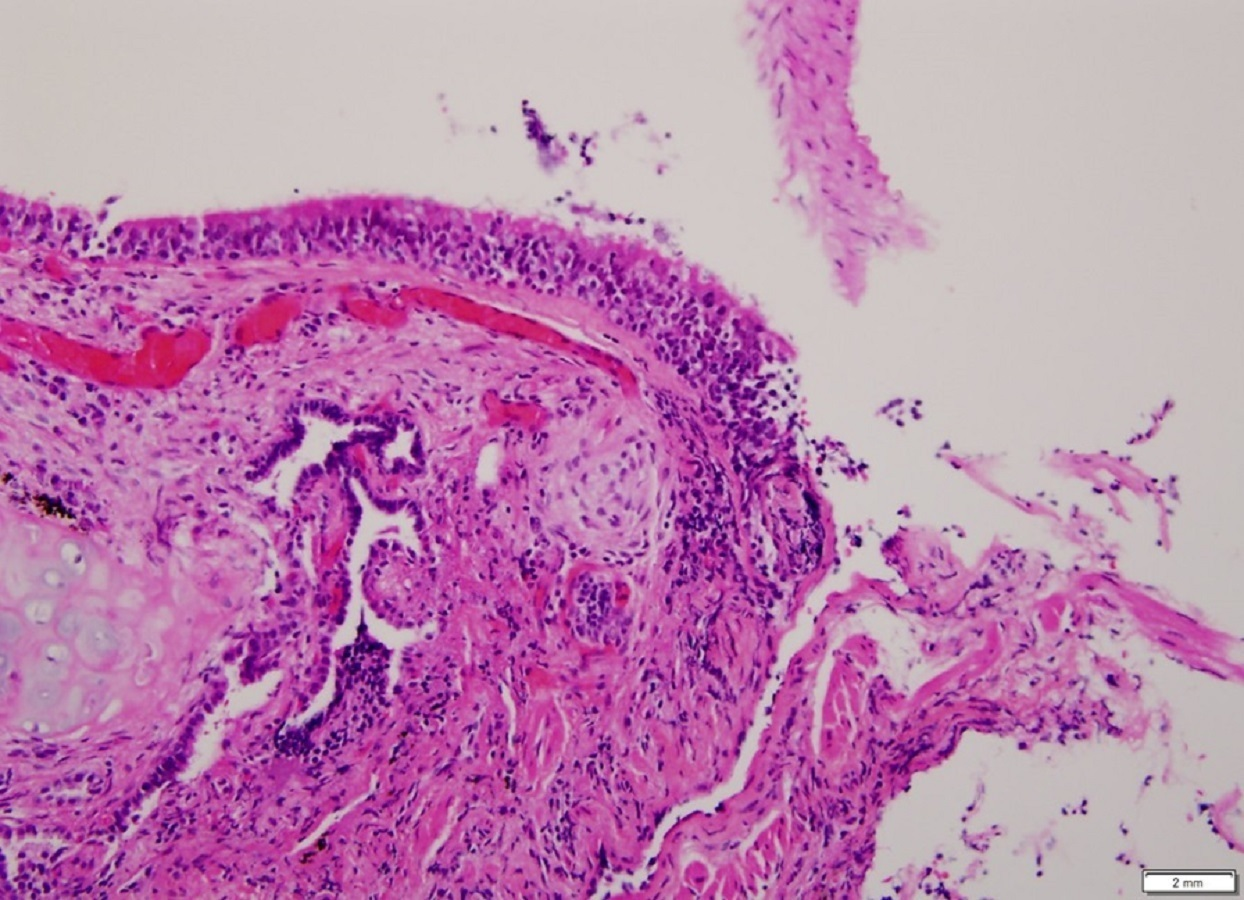
Transbronchial biopsy demonstrating a poorly formed, noncaseating granuloma

A chest x-ray was obtained which revealed bilateral nodular opacities. Subsequently, a computed tomography (CT) of the chest was obtained which showed subcentimeter cavitary nodular lesions (Figure 1). Respiratory culture, antinuclear antibodies, and antineutrophil cytoplasmic antibodies were negative. Thereafter, the patient underwent bronchoscopy. Broncho-alveolar lavage showed 40% macrophages and 30% lymphocytes with no malignant or Langerhan’s cells. A transbronchial biopsy culture came back positive for Mycobacterium avium complex (MAC), and the patient was started on antimycobacterial therapy for treatment of the MAC infection. At the three month follow-up, she reported worsening of her symptoms. A repeat CT chest demonstrated the development of more cavitary lesions (Figure 2) and mediastinal lymphadenopathy (Figure 3). A repeat bronchoscopy with endobronchial ultrasound was performed. The transbronchial biopsy showed a small, poorly formed non-caseating granuloma (Figure 4) and fine-needle aspiration of the lymph nodes revealed small lymphocytes with no malignant cells or granulomas.

 

**Figure 5:**
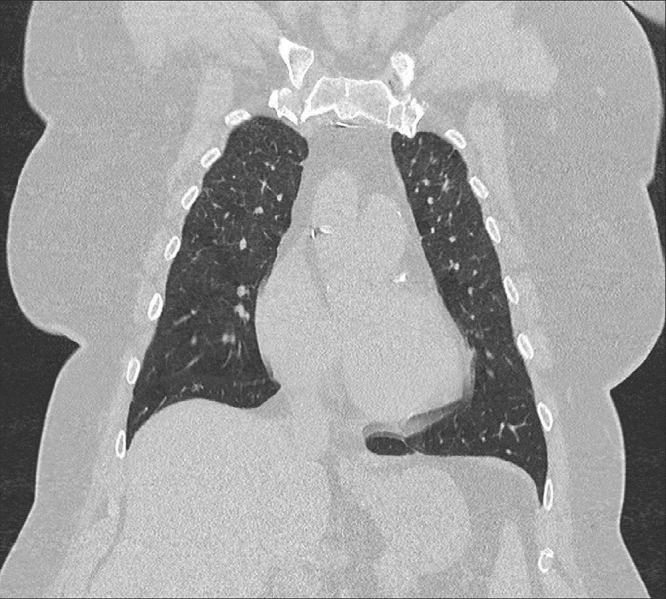
Computed tomography of the chest repeated after avoidance of hot tub and treatment with systemic steroids demonstrating significant improvement

The initial diagnosis was revisited and further history obtained from the patient revealed the regular use of a hot tub at home. She was advised complete avoidance of the hot tub. At one month follow-up, she reported significant improvement in her symptoms and a diagnosis of HTL was made. Antimycobacterial therapy was stopped and a six-week course of systemic steroids was initiated. Thereafter, there was continued improvement in her clinical symptoms, and repeat imaging demonstrated complete resolution of the cavitary lesions and lymphadenopathy (Figure 5).

## Discussion

HTL is a diffuse granulomatous lung disease that is associated with exposure to MAC and other NTMs that contaminate the hot tub water, swimming pools, and household showers [3-4]. Predisposing risk factors identified include poor compliance with hot tub maintenance, and inadequate ventilation and aerosolization. Its pathogenesis continues to be a matter of debate as it exhibits both infectious and hypersensitivity-like features. However, the majority of the literature thus far supports the hypersensitivity hypothesis [1, 3, 5]. Clinically, it manifests as an acute flu-like illness with cough, fever, joint pain followed by protracted symptoms, such as dyspnea on exertion, fatigue, and weight loss [6]. Diagnostic criteria for HTL include the sub-acute onset of respiratory symptoms associated with hot tub exposure, positive mycobacterial cultures from respiratory and water samples, and characteristic radiographic findings [1].

Thus far, less than 100 cases have been described in the literature and the only radiographic findings reported are diffuse centrilobular nodular and/or ground glass opacities [2]. In our patient, it presented as cavitary nodular opacities which were suggestive of a MAC infection [7]. However, the patient’s clinical and radiographic status worsened despite being on antimycobacterial therapy for an adequate length of time, and significant improvement was only achieved with avoidance of hot tub exposure, which is a hallmark characteristic of hypersensitivity pneumonitis (HP).      

Another interesting radiographic finding in our case was the development of mediastinal lymphadenopathy that complicated the clinical picture even further as it has not been reportedly seen with HTL [6]. Our suspicion for sarcoidosis was allayed when the transbronchial biopsy showed a poorly formed, noncaseating granuloma which is typically seen with HP [8-9], the absence of granulomas on fine-needle aspiration of the mediastinal lymph nodes, a low CD4/CD8 ratio, and a significant improvement in symptoms with just avoidance of the hot tub. 

Treatment of HTL is the same as for any other type of HP and primarily rests on avoidance of exposure to hot tubs. Those with severe disease can be given a trial of corticosteroid therapy. Though antimycobacterial therapy may be used if the above measures fail, there are currently no data to say which patients would benefit from it [6]. There is usually recovery without relapse with the above-outlined treatment plan. So far, no deaths have been reported regardless of the delay in diagnosis or severity of the disease at presentation.     

## Conclusions

Our case further endorses the hypothesis that HTL is a hypersensitivity-like pneumonitis rather than an infectious process. We also believe that this is the first reported case of HTL that manifested radiographically as cavitary nodular lung lesions with mediastinal lymphadenopathy. We, hereby, recommend that physicians should consider HTL as a possible etiology when encountered with these radiographic findings.  
